# Association between Club Sports Participation and Physical Fitness across 6- to 14-Year-Old Austrian Youth

**DOI:** 10.3390/ijerph16183392

**Published:** 2019-09-12

**Authors:** Clemens Drenowatz, Klaus Greier, Gerhard Ruedl, Martin Kopp

**Affiliations:** 1 Division of Physical Education, University of Education Upper Austria, 4020 Linz, Austria; 2 Division of Physical Education, Private University of Education (KPH-ES), 6422 Stams, Austria; nikolaus.greier@kph-es.at; 3 Department of Sport Science, University of Innsbruck, 6020 Innsbruck, Austria; Gerhard.Ruedl@uibk.ac.at (G.R.); Martin.Kopp@uibk.ac.at (M.K.)

**Keywords:** cardiorespiratory endurance, strength, power, agility, body weight, children, adolescents, physical activity

## Abstract

Changes in social and built environments most likely contribute to a decline in physical activity (PA) and physical fitness in children and adolescents. Organized sports may be an important component in ensuring adequate fitness, which is an important aspect in general health and well-being. The present study examines differences by club sports participation in cardiorespiratory endurance, muscular strength, power, speed, agility, flexibility and balance in 3293 (55.1% male) Austrian children and adolescents between 6 and 14 years of age. Anthropometric measurements (height and weight) were taken and participants completed the German motor test during regular class time. Even though there was no significant difference in body weight between club sports participants and non-club sports participants, club sports participation was associated with higher physical fitness, particularly regarding endurance, strength, power, and agility. Differences by club sports participation, however, declined during the elementary school years (6–10 years of age), while they became more pronounced during middle school years (10–14 years of age). Club sports participation, therefore, may be a viable option in the promotion of physical fitness, particularly during adolescence. At younger ages, other sources of PA, such as physical education and free play, however, should be considered to ensure sufficient fitness levels that contribute to a healthy and active lifestyle.

## 1. Introduction

Insufficient physical activity (PA) is one of the major threats to public health due to the detrimental effects on physical and mental health [1,2]. Of particular concern is the high number of youth who are not meeting the current recommendation of at least 60 min of moderate-to-vigorous PA per day [3,4,5], as habits and attitudes towards PA are formed at young ages and are subsequently carried into adulthood [6]. Many children and adolescents already present at least one cardiometabolic risk marker [7], even though clinical symptoms of cardiovascular disease (CVD) may not become apparent until adulthood [8]. The decline in PA at young ages has at least partially been attributed to changes in social and built environments, which facilitate an increasingly sedentary lifestyle of children and adolescents [9,10,11]. Besides prolonged sitting times during school hours, sedentary pursuits such as watching TV or playing computer games have become popular leisure choices [12,13]. Low PA levels during childhood and adolescence have also contributed to a decline in physical fitness [14,15,16], which has detrimental effects on various health outcomes and affects cognitive development and academic performance [17,18,19]. Physical fitness is defined as a person’s ability to carry out daily activities without undue fatigue and adequate energy reserves to enjoy recreational pursuits [20]. Physical fitness, therefore, may also be an important contributor to an active and healthy lifestyle beyond childhood and adolescence.

Given the large amount of sedentary choices during leisure time, which hinders recreational PA, participation in organized PA such as club sports is becoming an important contributor to PA in youth [21,22,23]. Additionally, organized sports have become one of the most popular contexts for health promotion in children and adolescents [4]. A recent review reported that 40–59% of European children and adolescents participate in club sports [4], and available evidence indicates that youth spend the majority of their daily PA in organized PA such as sports [24,25]. Accordingly, club sports participation has been associated with greater total PA and a higher probability of meeting PA recommendations [22,26,27,28]. Particularly, recommendations for vigorous PA have been more definitively achieved in club sports participants [28,29], and there appears to be a beneficial association of sports participation during childhood and adolescence with PA levels during adulthood [30,31,32]. 

Sports participation has also been shown to have a stronger relationship with physical fitness compared to non-organized leisure time PA [33]. This may at least partially be attributed to the energetic challenges of club sports participation, as habitual PA bursts may not be of sufficient volume and intensity to modify physical fitness [34,35]. Higher fitness may further contribute to a greater enjoyment of PA and increase the likelihood for a sustainable active lifestyle. In addition, physical fitness has been suggested as an important biomarker for health and well-being [36]. In order to enhance the understanding of the association between club sports participation and physical fitness that could inform intervention strategies, additional research is warranted. The present study, therefore, examines differences in cardiorespiratory endurance, strength, power, speed, agility, flexibility and balance by club sports participation across a wide age range of Austrian children and adolescents.

## 2. Materials and Methods 

A total of 20 elementary schools (grades 1–4) and 14 middle schools (grades 5–8) from various districts in Western Austria were contacted for participation in the study. Six schools (two elementary schools, four middle schools) declined to participate due to organizational problems resulting in a final sample of 28 schools with 3293 (55.1% male) participants. Data was collected between September 2015 and December 2016. Parents received written information about the study prior to data collection and provided written informed consent. Oral assent was obtained from the participants at the time of data collection. The study was conducted in accordance with the declaration of Helsinki 1975, revised in 2013 (https://www.wma.net/what-we-do/medical-ethics/declaration-of-helsinki/), and was approved by the Institutional Review Board of the University of Innsbruck, Austria and the respective school boards. 

### 2.1. Anthropometric Measurements 

Body weight (kg) and height (cm) were measured according to standard procedures with children wearing gym clothes and in bare feet. Body weight was measured to the nearest 0.1 kg with an electronic scale (GRUNDIG 3710, Grundig AG, Germany), and height was measured to the nearest 0.1 cm with a portable stadiometer (SECA 217, Seca, Germany). Body mass index (BMI) was calculated (kg/m²) and converted to BMI percentiles (BMIPCT) based on German reference values [37]. Subsequently, participants were classified as underweight (<10th percentile), normal weight (10–90th percentile) or overweight/obese (>90th percentile). 

### 2.2. Physical Fitness and Sports Participation

Physical fitness was assessed with the German motor test (Deutscher Motorik Test, DMT6-18) [38]. The DMT6-18 is a standardized and validated test that consists of eight items (20 m sprint, standing long jump, sit ups, push-ups, 6-min run, sideways jumping, backwards balancing, stand-and-reach test) and provides information on speed, power, strength, cardiorespiratory endurance, agility, balance, and flexibility. All tests were administered by trained personnel and completed in a single session during regular school time. After a standardized warm-up, participants performed the 20 m sprint. The remaining tests were performed in random order, except for the 6-min run, which was performed at the end of the test session. In addition to raw performance values, the DMT6-18 provides age- and sex-standardized scores based on a German reference sample [37], with a score of 100 indicating average performance for the respective age and sex. The mean of the age- and sex-standardized scores across all test items was further used as the overall fitness score.

Sports participation was determined via self-report, and students were classified as either club sports participants or non-club sports participants.

### 2.3. Statistical Analysis

Descriptive statistics were calculated and are reported as mean with standard deviation for interval-scaled data and prevalence for nominal data. A 2 (club sports participation) by 9 (age) ANOVA was used to examine the association of club sports participation and age with BMIPCT and overall physical fitness. In cases of significant interaction effects, differences across age groups were examined via ANOVA separately for club sports participants and non-participants. In addition, a 2 × 9 MANOVA was used to examine the association of club sports participation and age with individual items of physical fitness using both raw performance scores as well as sex- and age-standardized values. In cases of significant interaction effects, MANOVA was used to examine differences across age groups separately for club sports participants and non-club sport participants. In order to control for the influence of body weight, similar analyses were performed with BMIPCT as covariate. All statistical analyses were performed with SPSS 24.0. The significance level was set at *p* < 0.05 with Bonferroni adjustment for multiple comparisons. 

## 3. Results

A total of 3293 (55.1% male) participants provided valid data. Table 1 provides an overview of the anthropometric characteristics by age group. Across the entire sample, 19.5% were classified as overweight/obese, with a significant increase in the prevalence of overweight/obesity across age groups (14.2% at 6 years to 22.2% at 14 years; *p* for trend = 0.001). Almost half of the participants (48.5%) reported club sports participation and there was no difference in club sports participation by age. Boys, however, reported higher club sports participation compared to girls (52.1% vs 44.1%; *p* < 0.001). 

**Figure 1:**
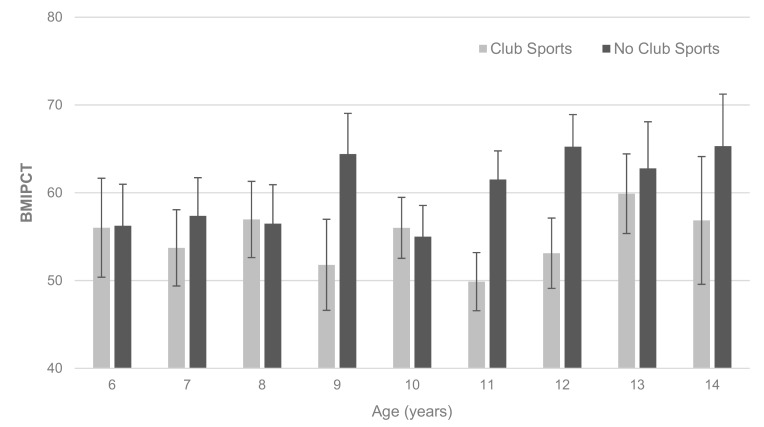
BMIPCT by age and club sports participation. Values are mean with 95% CI.

There was a significant interaction effect of age and club sports participation on BMIPCT (F(8, 3274) = 3.732; *p* < 0.001). Among club sports participants, BMIPCT remained relatively stable across age groups (*p* for trend = 0.576), while BMIPCT increased with increasing age in non-club sports participants (*p* for trend = 0.001). Across the entire sample, club sports participation was associated with a lower BMIPCT (*p* < 0.001), with the difference becoming more pronounced after the age of 10 years (Figure 1).

**Figure 2:**
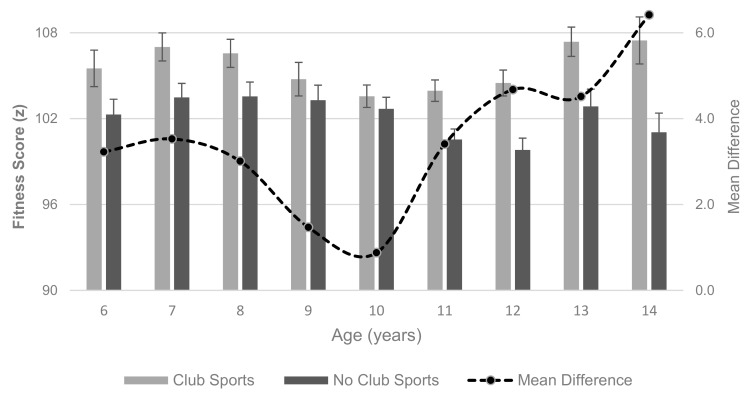
Total fitness score by age and club sports participation. Values are mean with 95% CI for bars and line indicates mean difference (mean club sports vs mean non-club sports).

As has been shown for BMIPCT, there was a significant interaction effect of age and club sports participation on physical fitness (F(8, 3275) = 4.838; *p* < 0.001). Using age- and sex-standardized fitness scores, non-club sports participants displayed a decline across age groups (*p* for trend < 0.001), while standardized fitness scores remained stable in club sports participants (*p* for trend = 0.422). Accordingly, physical fitness was higher in club sports participants compared to non-club sports participants across the entire sample (total fitness score of 105.2 ± 6.7 vs 102.0 ± 6.9; *p* < 0.001). Figure 2 further shows that differences between club sports and non-club sports participants in physical fitness declined during the elementary school years (ages 6–10 years) and then increased during middle school years (ages 10–14 years). These results remained essentially unchanged after adjusting for BMIPCT.

Regarding individual components of physical fitness, absolute performance improved significantly with increasing age across all test items (*p* for trend < 0.001), except for the stand and reach test. Nevertheless, there were significant age-by-club sport participation interaction effects for sprint (F(8, 3268) = 2.716; *p* = 0.006), sideways jumping (F(8, 3268) = 2.454; *p* = 0.012), standing long jump (F(8, 3268) = 5.759; *p* < 0.001), push-ups (F(8, 3268) = 4.122; *p* < 0.001), sit ups (F(8, 3268) = 2.676; *p* = 0.006) and the 6-min run (F(8, 3268) = 5.812; *p* < 0.001). Across the entire sample, club sports participation was associated with better performance on these test items (*p* ≤ 0.002), with differences between club sports participants and non-club sports participants becoming more pronounced after the age of 10 years (Table 2). No significant interaction effects were observed for the balance and stand-and-reach test. All results remained essentially unchanged after adjusting for BMIPCT. 

**Figure 3:**
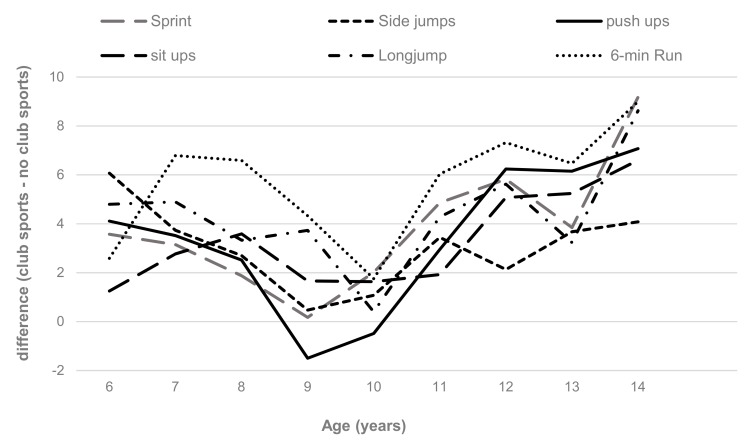
Mean difference in age- and sex-standardized scores between club sports and non-club sports participants on individual fitness tasks across the ages 6–14 years.

When age- and sex-standardized values were used, significant club-sports-by-age interaction effects were observed for sprint (F(8, 3268) = 3.279; *p* < 0.001), sideways jumping (F(8, 3268) = 2.234; *p* = 0.022), standing long jump (F(8, 3268) = 3.848; *p* < 0.001), push-ups (F(8, 3268) = 4.795; *p* < 0.001), sit ups (F(8, 3268) = 3.308; *p* = 0.001) and the 6-min run (F(8, 3268) = 5.996; *p* < 0.001). Sprint and push-up performance increased in club sports participants across age groups (*p* for trend ≤ 0.006), while it remained stable in non-club sports participants (*p* for trend ≥ 0.327). Long-jump and sit up performances remained stable across age groups in club sports participants (*p* for trend ≥ 0.599), while there was a decline in non-club sports participants for these test items (*p* for trend ≤ 0.005). Even though sideways jumping was stable across age groups in club sports participants and non-club sports participants (*p* for trend ≥ 0.618) and endurance performance declined across age groups in both groups (*p* for trend < 0.001), differences in sex- and age-standardized performance between club sports and non-club sports participants became more pronounced after the age of 10 (Figure 3). Between the ages of 6 and 10 years, differences between club sports and non-club spots participants became, in general, less pronounced. Nevertheless, club sports participants displayed better performance in all test items compared to non-club sports participants across the entire sample (*p* ≤ 0.002). All results remained essentially unchanged after adjusting for BMI PCT.

## 4. Discussion

The present study examined differences in various components of physical fitness and body weight by club sports participation in children and adolescents. Consistent with previous studies, absolute performance on fitness tests increased with increasing age [34,39], which can be attributed to increased body size, changes in body composition and neuromuscular maturation [40]. The differences between club sports and non-club sports participants, however, further indicated beneficial associations of club sports participation with physical fitness, particularly regarding endurance, strength, power and agility. Differences in balance and flexibility, on the other hand, were limited between club sports and non-club sports participants. Even though club sports participation was also associated with lower body weight, these results were independent of body weight. Of additional interest was that differences in physical fitness between club sports participants and non-club sports participants became particularly pronounced during middle school years (10–14 years of age) while differences in physical fitness declined during elementary school years (6–10 years of age). 

The beneficial association of club sports with physical fitness has been shown in previous studies [41,42,43,44]. Several training studies further showed adaptations in myocardial structure and function that resulted in improved cardiorespiratory fitness [45,46]. The present study, however, indicated that this association varies by age. Based on the results of this study, it can be argued that the contribution of club sports participation to physical fitness is limited during the elementary school years. This may be attributed to lower training loads during club sports at young ages. Further, physical education (PE) and a greater drive for free play may help to mitigate the differences between club sports and non-club sports participants. The importance of the school as an intervention setting targeting PA has also been emphasized by various studies that have shown an increase in PA during and after school hours [47,48]. During adolescence, PE time may be reduced due to other academic demands, and PA levels in general have been shown to decline at an estimated 7% per year with increasing age [49]. More structured approaches such as club sports, therefore, could become an increasingly important stimulus for the development of physical fitness. In addition, participation in club sports has been associated with more time spent in active free play [50], which may further contribute to adaptive responses and associated health benefits [17]. Due to the physical demands of many sports, club sport participation appears to particularly affect the development of endurance, strength, power and agility. Participation in ball games in particular has been associated with improved fitness due to the high amount of vigorous PA along with the versatility of training [41,45,51]. Given the association between club sports participation and vigorous PA [52,53], Koorts et al. have argued that club sports could have considerable long-term effects, even in the absence of significant contributions to total PA [54]. 

Ensuring sufficient time for engagement in vigorous PA during childhood and adolescence may be of particular importance, as results of the present study indicate an endurance capacity that is already declining at young ages. Endurance capacity, however, is an important component in minimizing CVD risk [55,56], and vigorous PA is more strongly related to CVD risk than total PA [57]. Given the positive impact on health, UK guidelines for children specifically recommend participation in vigorous PA at least three times per week in addition to the generally recommended 60 min of moderate-to-vigorous PA per day [58]. Participation in vigorous PA and club sports has further been associated with a lower body fat percentage and higher lean body mass [41,52], even though the association between club sports participation and body weight remains inconclusive [27,59]. In order to affect body weight, a certain amount of sports participation appears to be necessary that may not be reached by all club sports participants [43,60]. The present study also shows limited differences in body weight between club sports and non-club sports participants. Further, it shows that higher fitness levels of club sports participants are independent of body weight. These results, however, may actually be encouraging, as they indicate that body weight does not discriminate between sports participation during childhood and adolescence. Nevertheless, club sport participation may affect weight management as results of the present study showed a stable BMIPCT across age groups in club sport participants, while BMIPCT increased across age groups in non-club sports participants. Accordingly, sports participation may affect body weight in the long-term and could contribute to the prevention of excess weight gain and becoming overweight or obese later in life. 

In addition to the beneficial association of sports participation with physical fitness and associated health benefits [17,61], club sports participation has been associated with various health behaviors such as lower screen time, healthier eating patterns and decreased substance abuse [52,62]. Accordingly, club sports participation has been suggested as a more-discriminatory indicator of a healthy lifestyle during childhood and adolescence than body weight [52]. Further, there is evidence of psychological and social benefits as a result of participating in sports that go beyond other forms of leisure time PA. Specifically, sports participation has been associated with higher emotional control, self-esteem, confidence and competence, as well as better social skills and fewer depressive symptoms [63]. In fact, it has been argued that participation in club sports, particularly in team sports, is an important component in the social development of children due to the interaction with coaches and peers [64,65]. These aspects of general health, however, were not examined in the present study.

There are also some other limitations of the present study that need to be considered when interpreting the results. Participants were stratified based on self-reported participation in club sports and there was no information on the type of sports and training they engaged in. Additionally, there was no information on how long participants have been engaged in club sports and on potential seasonal differences in sports participation. Accordingly, participants reporting club sports may not have been involved in active training at the time of the fitness assessment. Further, there was no objective assessment of total PA or information on other forms of PA, which may affect physical fitness. Club sports participants may have also been more familiar with fitness testing and may have given a greater effort at the individual tests, which could have affected the results. Finally, the cross-sectional nature of the study does not allow causality to be established. While sports participation potentially enhances physical fitness, it is also possible that children and adolescents with higher fitness levels are more likely to participate in club sports. The large sample size and wide age range, on the other hand, was a considerable strength of this study. Similar fitness tests have been used across the entire age range, and the test battery has been shown to be valid and reliable across the study population [38]. An additional benefit of the DMT6-18 is the availability of age- and sex-standardized performance scores, which allows fitness levels to be evaluated across different age groups while accounting for growth related changes.

In summary, the present study shows beneficial associations between participation in organized sports and physical fitness during childhood and adolescence. As physical fitness has been considered a powerful marker of health [17], club sports could be a key component in the promotion of an active and healthy lifestyle, particularly in youth [28]. Accordingly, an expert panel recently emphasized the value of sports to children and adolescents from a prevention perspective due to the range of health benefits [66]. If clubs sports are considered to be an important modifiable determinant in public health, non-club sports participants, however, may warrant special attention [28]. In order to specifically target at-risk populations, a better understanding of the correlates of club sport participation, such as social class, ethnicity and sex, is needed. Further, other approaches, including PE, may need to be considered. The present study indicates the potential benefits of PE in the development of fitness, particularly during childhood, as differences in physical fitness between club sports participants and non-club sports participants declined during the elementary school years. Most likely, a combination of in-school and out-of-school interventions (e.g., PE, club sports, free play space) will provide the best strategy for the promotion of PAs that enhance physical fitness in youth and contribute to sustainable beneficial changes in lifestyle characteristics, which facilitate an active and healthy lifestyle beyond childhood and adolescence.

## 5. Conclusions

In light of the changes in social and built environments that contribute to an increasingly sedentary lifestyle in children and adolescents, organized sports appears to be an important contributor to ensuring sufficient physical fitness in youth. Given the association between physical fitness and general health and well-being, this could also have important health implications later in life.

## Figures and Tables

**Table 1:** Anthropometric characteristics by age group. Values are mean ± SD.

Age Group (N, % Male)	Height (cm)	Weight (kg)	BMI PCT
6 years (N = 254, 55.9%)	121.8 ± 6.1	24.1 ± 4.7	56.1 ± 28.3
7 years (N = 354, 52.3%)	126.5 ± 5.8	26.5 ± 5.1	55.5 ± 27.9
8 years (N = 347, 52.7%)	131.5 ± 6.7	29.8 ± 6.4	56.7 ± 29.6
9 years (N = 279, 52.3%)	137.2 ± 6.7	33.8 ± 7.8	58.8 ± 29.7
10 years (N = 542, 55.7%)	144.2 ± 7.5	37.9 ± 9.1	55.5 ± 30.7
11 years (N = 620, 55.5%)	149.0 ± 7.9	42.2 ± 11.0	55.8 ± 30.3
12 years (N = 459, 57.7%)	156.5 ± 7.3	49.3 ± 12.5	59.8 ± 30.5
13 years (N = 280, 57.2%)	161.4 ± 7.9	54.1 ± 12.9	61.1 ± 29.1
14 years (N = 158, 55.7%)	164.5 ± 8.3	58.2 ± 12.8	61.9 ± 28.6

BMIPCT: BMI percentile based on German reference values [37].

**Table 2:** Physical fitness in club sports participants and non-club sports participants by age group. Values are mean ± SD.

		6 Years	7 Years	8 Years	9 Years	10 Years	11 Years	12 Years	13 Years	14 Years
**Sprint (sec)**	*Club*	4.8 ± 0.5	4.5 ± 0.4	4.4 ± 0.4	4.3 ± 0.4	4.2 ± 0.3	4.0 ± 0.4	3.8 ± 0.4	3.7 ± 0.3	3.6 ± 0.3
	*No Club*	5.0 ± 0.6	4.7 ± 0.4	4.5 ± 0.5	4.3 ± 0.4	4.3 ± 0.4	4.2 ± 0.4	4.0 ± 0.4	3.8 ± 0.4	3.9 ± 0.4
**Side Jump (reps)**	*Club*	24.3 ± 5.5	28.5 ± 6.9	33.8 ± 7.1	35.1 ± 7.6	36.4 ± 6.5	40.4 ± 7.7	43.6 ± 7.7	48.6 ± 7.4	48.8 ± 5.6
	*No Club*	21.7 ± 6.2	25.7 ± 6.0	31.4 ± 7.3	34.6 ± 7.3	35.6 ± 6.5	37.8 ± 7.4	41.2 ± 6.7	44.4 ± 7.0	44.3 ± 5.9
**Long Jump (cm)**	*Club*	116.5 ± 17.4	124.9 ± 18.0	131.1 ± 19.7	137.2 ± 19.7	144.6 ± 21.0	156.2 ± 21.5	164.5 ± 26.5	171.0 ± 25.8	184.8 ± 27.4
	*No Club*	107.1 ± 18.3	114.9 ± 18.9	123.3 ± 18.9	128.6 ± 21.5	144.2 ± 22.7	146.2 ± 22.5	141.1 ± 22.5	164.1 ± 30.9	159.2 ± 29.9
**Push-Ups (reps)**	*Club*	12.2 ± 4.1	14.3 ± 4.6	15.9 ± 4.2	14.8 ± 4.4	14.7 ± 6.5	15.6 ± 5.9	16.5 ± 5.9	19.3 ± 4.2	19.7 ± 4.6
	*No Club*	10.7 ± 3.9	12.7 ± 4.3	14.9 ± 4.8	15.3 ± 4.2	14.9 ± 6.2	14.4 ± 6.0	14.1 ± 5.6	17.1 ± 5.2	16.5 ± 3.4
**Sit Ups (reps)**	*Club*	14.6 ± 5.7	18.2 ± 5.0	21.2 ± 4.9	21.6 ± 5.4	21.8 ± 6.2	22.8 ± 6.3	24.5 ± 5.9	27.5 ± 6.3	28.1 ± 5.7
	*No Club*	13.4 ± 5.7	16.2 ± 5.6	18.5 ± 5.3	20.5 ± 5.3	20.9 ± 6.3	21.4 ± 6.4	21.2 ± 5.4	24.8 ± 6.2	24.4 ± 5.1
**6-Min Run (m)**	*Club*	877 ± 138	950 ± 132	963 ± 133	956 ± 163	987 ± 161	1056 ± 158	1084 ± 185	1092 ± 178	1107 ± 167
	*No Club*	831 ± 149	855 ± 130	871 ± 136	890 ± 124	968 ± 154	959 ± 157	962 ± 177	991 ± 172	943 ± 165
**Ba-lance (steps)**	*Club*	25.5 ± 9.3	31.1 ± 9.2	34.3 ± 9.7	36.0 ± 8.4	37.8 ± 9.1	38.6 ± 8.4	39.9 ± 7.9	41.3 ± 7.8	41.9 ± 7.2
	*No Club*	25.0 ± 9.7	28.4 ± 10.0	31.3 ± 9.1	35.8 ± 9.0	35.7 ± 9.8	36.6 ± 8.8	36.8 ± 8.8	37.1 ± 9.6	37.0 ± 8.2
Stand & Reach (cm)*	*Club*	1.7 ± 4.7	1.5 ± 6.0	0.6 ± 7.5	–0.1 ± 6.5	–0.7 ± 7.5	–0.7 ± 7.7	0.2 ± 8.4	2.7 ± 9.3	4.0 ± 8.9
	*No Club*	1.2 ± 6.2	1.8 ± 6.1	0.3 ± 6.7	–1.3 ± 7.4	–1.3 ± 6.9	–1.4 ± 7.2	–1.3 ± 8.8	1.4 ± 9.5	1.6 ± 9.9

* Negative values indicate poorer performance (not reaching toes).
